# Targeting Canonical Wnt-signaling Through GSK-3β in Arrhythmogenic Cardiomyopathy: Conservative or Progressive?

**DOI:** 10.1007/s12265-024-10567-x

**Published:** 2024-10-11

**Authors:** Brandon Shu Huang Low, Angeliki Asimaki

**Affiliations:** 1 College of Medical and Dental Sciences, University of Birmingham, Birmingham, UK; 2 Cardiovascular and Genomics Research Institute, City St. George’s, University of London, London, UK; 3 Cardiovascular Clinical Academic Group, City & St George’s University of London, Cranmer Terrace, London, SW17 0RE UK

**Keywords:** Canonical Wnt-signaling, Glycogen synthase kinase 3β, Hippo, Arrhythmogenic cardiomyopathy, SB216763, CHIR99021, Tideglusib

## Abstract

**Abstract:**

Arrhythmogenic cardiomyopathy is a primary myocardial disease and a major cause of sudden death in all populations of the world. Canonical Wnt signalling is a critical pathway controlling numerous processes including cellular differentiation, hypertrophy and development. GSK3β is a ubiquitous serine/threonine kinase, which acts downstream of Wnt to promote protein ubiquitination and proteasomal degradation. Several studies now suggest that inhibiting GSK3β can prevent and reverse key pathognomonic features of ACM in a range of experimental models. However, varying concerns are reported throughout the literature including the risk of paradoxical arrhythmias, cancer and off-target effects in upstream or downstream pathways.

**Clinical Relevance:**

In light of the start of the phase 2 TaRGET clinical trial, designed to evaluate the potential therapeutic efficacy of GSK3β inhibition in patients with arrhythmogenic cardiomyopathy, this report aims to review the advantages and disadvantages of this strategy.

**Graphical Abstract:**

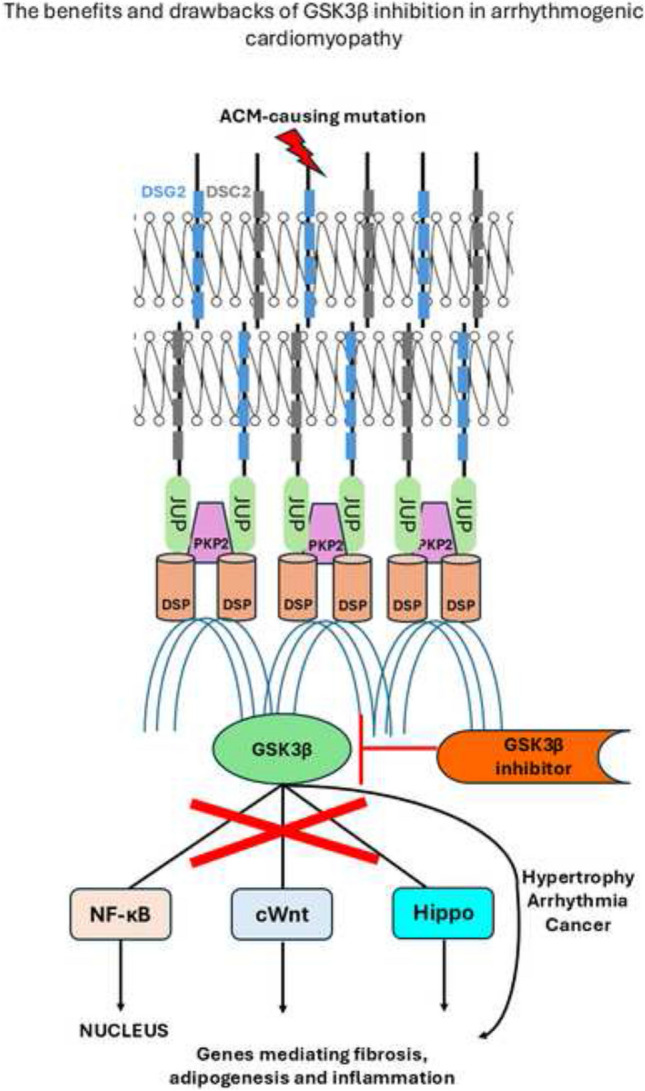

## Introduction

Arrhythmogenic cardiomyopathy (ACM) is a primary myocardial condition characterized clinically by ventricular arrhythmias and sudden cardiac death (SCD) and pathologically by the gradual degeneration of cardiac myocytes (CMs) and their subsequent replacement by fat and fibrous tissue [1]. ACM affects approximately 1:1000–1:5000 individuals in the general population, with young adults and athletes at higher risk of SCD [1]. Etiology is mainly linked to mutations in genes encoding desmosomal proteins. Desmosomes are protein adhesion complexes, residing in the IDs, the areas that mechanically and electrically couple CMs [2]. It is thought that desmosomal gene mutations promote ACM development through weakening CM adherence and aberrant activation of mechano-transduction pathways [2]. Despite decades of research, management approaches remain merely symptom-targeting. The first line of management includes anti-arrhythmic medication and ICDs, which, however, do not prevent disease progression. Accordingly, mechanism-based approaches are urgently required.

Multiple lines of evidence now suggest that inhibition of GSK3β can ameliorate disease development in a wide range of ACM experimental models [3, 4]. However, concerns exist surrounding potential off-target effects. In light of the current TaRGET trial, designed to assess the therapeutic efficacy of the GSK3β inhibitor tideglusib in patients with ACM [5], it is of pivotal importance to review the literature on the advantages and disadvantages of this mechanism-based approach.

**Fig. 1:**
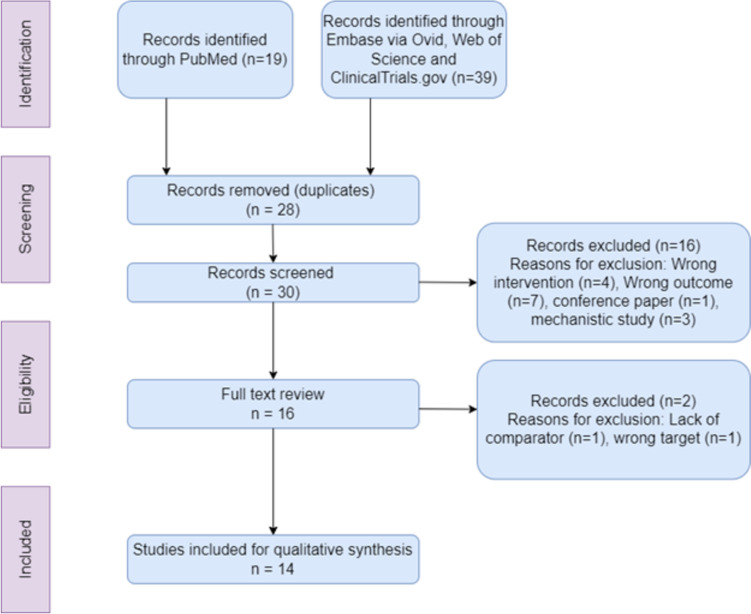
Flowchart of study selection methodology

All publications up to 2024 were systematically selected via electronic databases for qualitative synthesis (Fig. 1). The following search queries were used: (arrhythmogenic cardiomyopathy OR arrhythmogenic right ventricular cardiomyopathy OR ARVC or ACM) AND (SB216763 OR GSK3β OR SB485232 OR tideglusib).

## Literature Review

### ACM

Early ACM is characterized by ventricular arrhythmias out of proportion to the degree of myocardial remodelling [1]. In later stages, the myocardium undergoes progressive degeneration and CMs are replaced by fibrofatty tissue [1]. Classical ACM affects primarily the right ventricle. More recent evidence, however, suggests that sole left ventricular and biventricular disease forms exist [1].

Over 60% of ACM patients bear one or more mutations in genes encoding for the cardiac desmosomal proteins: *DSP*, *PKP2*, *DSG2*, DSC2 and *JUP* [1]. Non-desmosomal gene mutations are also associated with ACM, where studies indicate the presence of a common final signaling pathway underlying disease pathogenesis (Table 1) [1].

**Table 1:** Table of genes associated with ACM, modified from Vallverdú-Prats et al. [6]

Gene	Protein	Frequency (%)
Desmosome		
*PKP2*	Plakophilin-2	19–46
*DSP*	Desmoplakin	1–16
*DSG2*	Desmoglein-2	2.5–10
*DSC2*	Desmocollin-2	1–8
*JUP*	Plakoglobin	rare
Adherens junction		
*CTNNA3*	αT-catenin	rare
*CDH2*	Cadherin 2	rare
*TJP1*	Tight junction protein ZO-1	rare
Cytoskeletal structure		
*LMNA*	Lamin A/C	rare
*DES*	Desmin	rare
*FLNC*	Filamin C	rare
*TMEM43*	Transmembrane protein 43	rare
*TTN*	Titin	rare
*ANK2*	Ankyrin B	rare
Ion transport		
*SCN5A*	Voltage-gated sodium channel	rare
*RYR2*	Ryanodine receptor 2	rare
*PLN*	phospholamban	rare
Cytokine		
*TGFβ3*	Transforming growth factor, beta-3	rare
Slicing factor		
*RBM20*	RNA-binding motif protein 20	rare

The table classifies the genes according to their function and provides information on the frequency that associated mutations are identified in cohorts of ACM patients

**Fig. 2:**
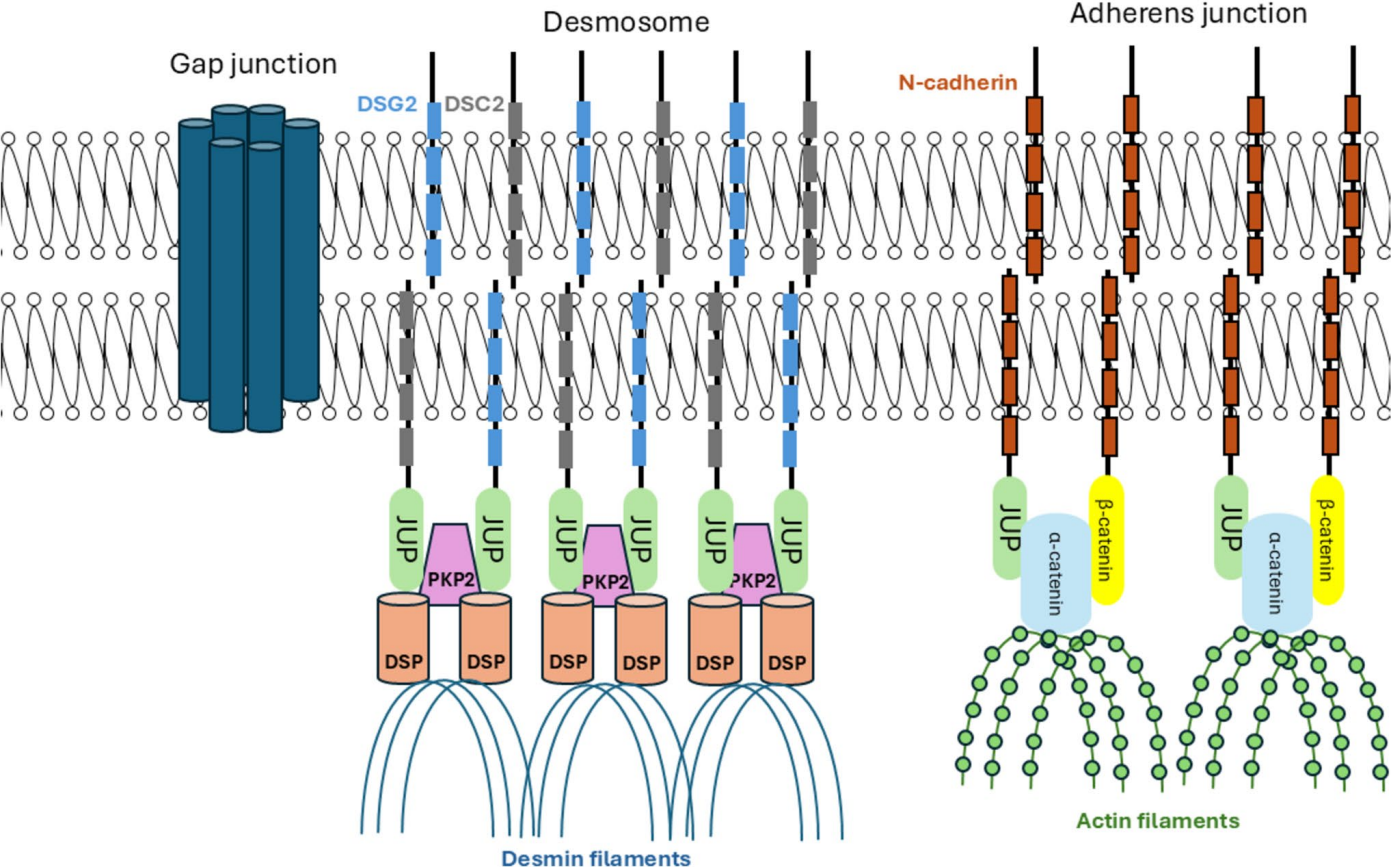
Diagrammatic representation of the cardiac ID. Among others, the multi-protein structure contains adherens junctions and desmosomes (involved in mechanical cell–cell adhesion) as well as gap junctions (involved in electrical propagation). Figure created with powerpoint

The cardiac ID involves 3 major protein complexes: AJs, GJs and desmosomes (Fig. 2). AJs anchor N-cadherin to the actin cytoskeleton via α- and β-catenin. Catenins have a dual role; both as adhesion molecules but also as transcriptional regulators [7]. GJs are comprised of connexins and are responsible for electrical conduction [7]. Desmosomes contain the desmosomal cadherins DSG2 and DSC2, which link adjacent CMs as well as the armadillo and plakin proteins DSP, PKP2 and JUP, which anchor the cadherins to the intermediate filaments [7]. IDs also contain ion channels, such as the voltage-gated sodium channels, responsible for action potential generation. The main protein subunit of the cardiac sodium channels is Nav1.5, coded by the *SCN5A* gene [7]. Multiple lines of evidence suggest that desmosomal gene mutations perturb the cWnt and Hippo signalling pathways, which in turn promote the fibrogenic and adipogenic phenotypes characterizing ACM [8, 9].

### Wnt/GSK3/Hippo Signaling

The highly conserved Wnt signaling pathway, originally recognized for its role in embryonic development and tissue homeostasis, has emerged as a crucial player in the pathogenesis of several human disorders and greatly contributes to disease progression with potential therapeutic implications [10]. CM differentiation from iPSCs is critically dependent upon Wnt regulation. Following initial Wnt activation, mesendoderm is generated. Thereafter, maintenance of Wnt signaling is critical to direct cell fate into cardiac mesoderm [11]. Wnt is comprised of canonical and non-canonical components. The canonical pathway is responsible for retaining the proliferative state of cardiac tissue during development and is an essential regulator of the expansion of mesenchymal cells populating the outflow tract cushions [12], whereas the non-canonical pathway primarily promotes precursor differentiation [13].

It is the canonical component that has been implicated in ACM pathogenesis [14, 15]. Efforts to target cWnt activation in experimental models have led to down-regulation of both Nav1.5 and the main ventricular gap junction protein Connexin43 (*GJA1*; Cx43) resulting in decreased cardiac conduction velocities [16]. Indeed, altered distribution and expression of both proteins is regarded as a phenotypic hallmark of ACM [17]. It is therefore, unsurprising that aberrant activation of cWnt may contribute to the pathogenesis of ACM.

**Fig. 3:**
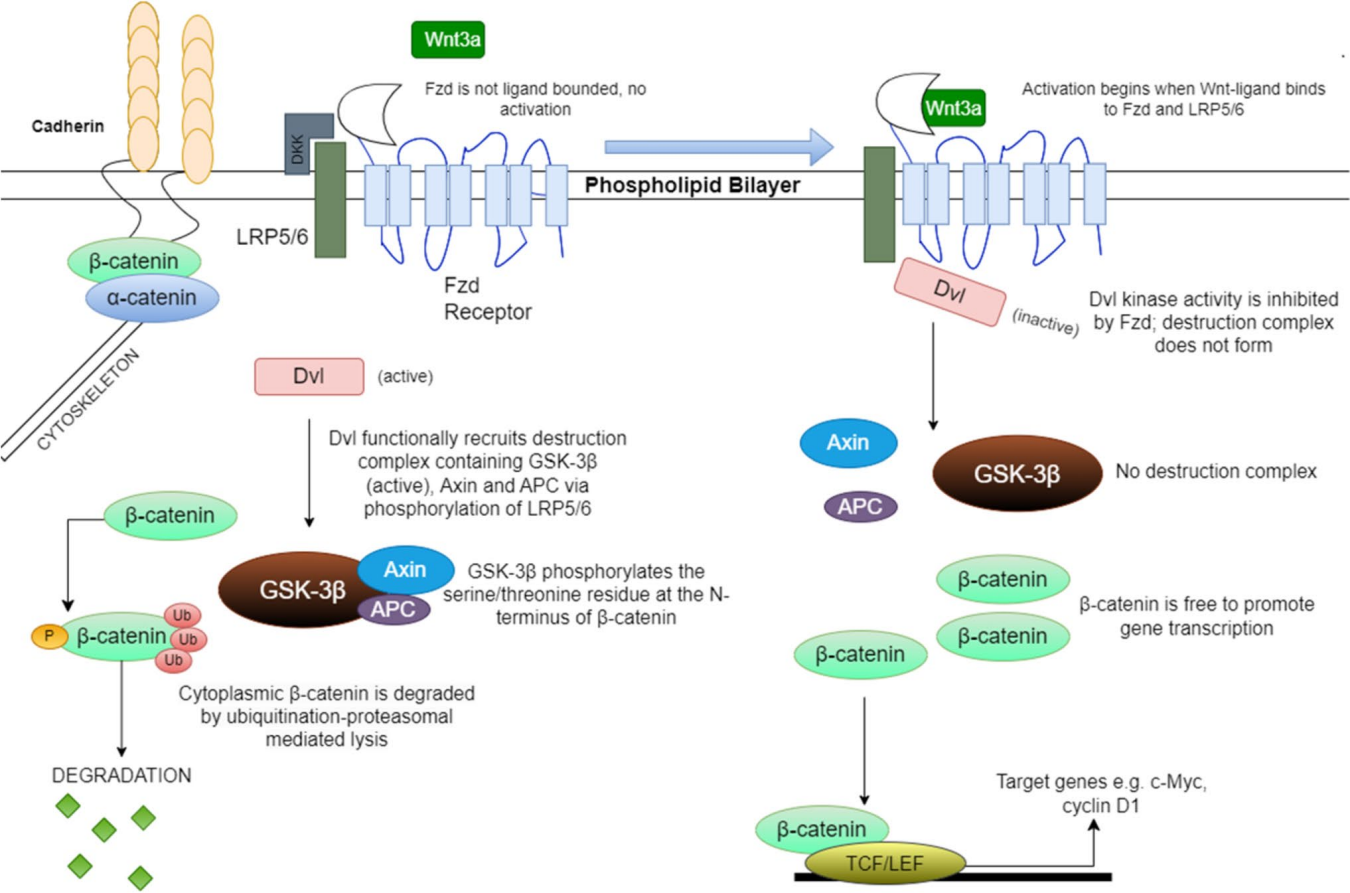
Representation of canonical Wnt signaling. **LEFT:** Upon binding of Wnt ligands to Fzd/LRP5/6 receptors, Dvl recruits GSK3β, APC and axin into the destruction complex. Active GSK3β phosphorylates serine/threonine residues on the N-terminal domain of β-catenin targeting it for ubiquitination and proteasomal degradation. **RIGHT:** In the absence of Wnt signals, Dvl activity is inhibited by Fzd. Accordingly, the destruction complex is not formed and β-catenin is free to enter the nucleus where it interacts with the TCF/LEF transcription factors to drive expression of target genes including c-Myc and Cyclin D1. Figure created with draw.io

**Fig. 4:**
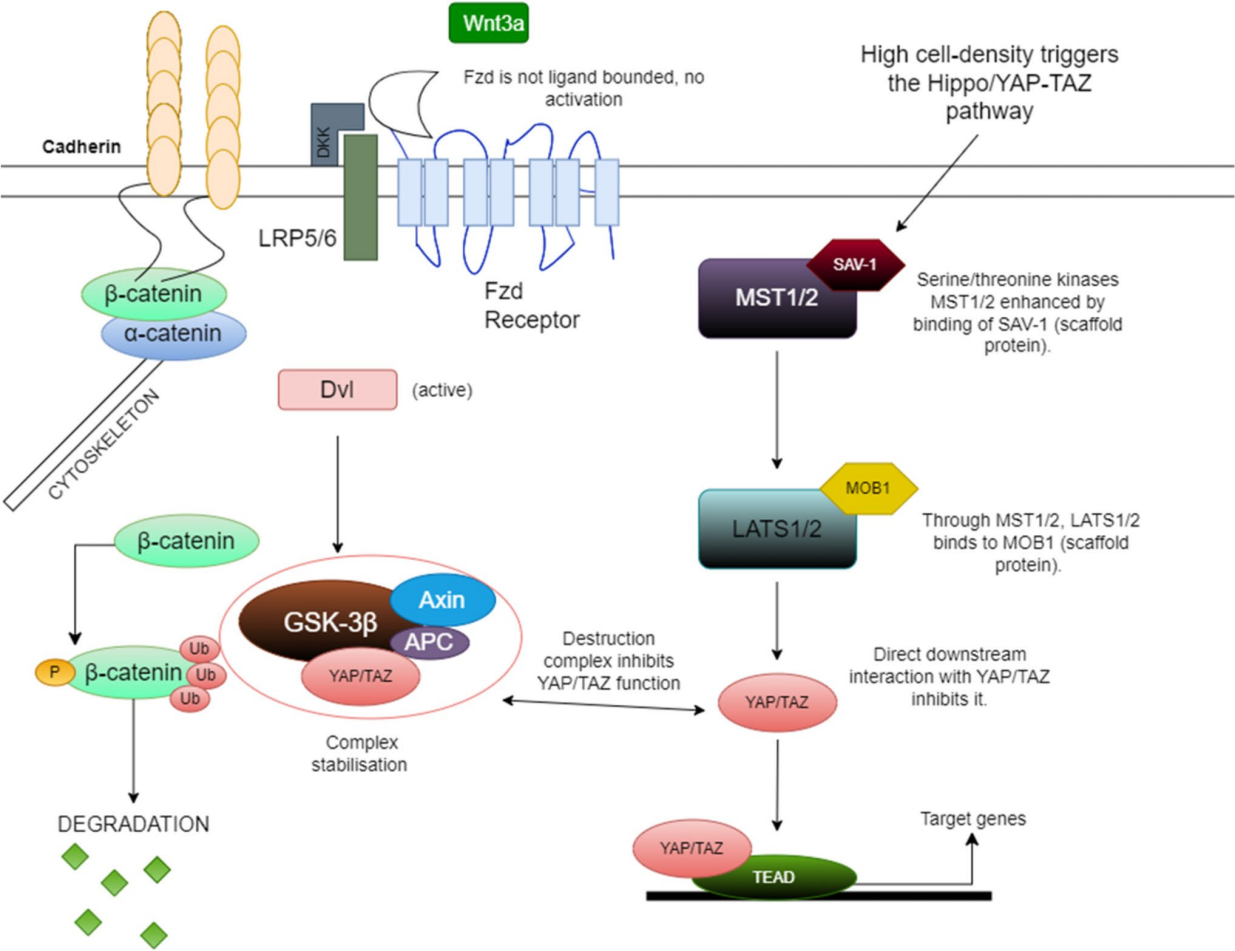
Representation of canonical Wnt/Hippo pathway crosstalk. Upon activation, MST1/2 phosphorylates LATS1/2 and its scaffold protein MOB1. Active LATS1/2 then phosphorylates and inactivates YAP/TAZ preventing it from translocating into the nucleus and binding to the TEAD transcription factors. There is bidirectional modulation and regulation between the pathways, where YAP/TAZ is inhibited by the Axin/APC/GSK-3β destruction complex. Figure created with draw.io

Glycogen synthase kinase-3 (GSK3) is a highly conserved serine/threonine protein kinase that is ubiquitously expressed as two isoenzymes; GSK3α and GSK3β. It was originally recognized for its ability to phosphorylate and inhibit glycogen synthase and hence promote insulin resistance. The ability of lithium to reverse this action led to its classification as a GSK3 inhibitor [18, 19]. Later, a class of maleimides (including SB2 and SB4) were shown to be more potent GSK3 inhibitors that act by competitively binding to the ATP-binding site [20, 21]. Wnt signaling regulates GSK3 activity by displacing GSK3 from its binding partners: axin and adenomatous polyposis coli (APC) in the so-called destruction complex. In the absence of Wnt ligand binding, β-catenin is phosphorylated by GSK3β and targeted for ubiquitination and proteasomal degradation. Upon binding to Frizzled/LRP5-6 receptors, Wnt ligands displace GSK3β precluding the degradation of β-catenin, which is then free to enter the nucleus and bind to the T cell factor (TCF)/lymphoid enhancer factor (LEF) transcription factors, leading to the transcription of Wnt target genes (Fig. 3) [22]. Expression of all Wnt1, GSK3β and β-catenin is significantly increased in the hearts of hypertensive rats of various aetiologies [23] while the ATP-competitive GSK3β inhibitor CHIR reverses pathological electrical remodelling in aged rats via restoring Nav1.5 and Cx43 levels at the cardiac IDs [24]. Evidence from patient hearts as well as murine and cellular ACM experimental models suggests that the Hippo pathway is also aberrantly activated in this disease [25]. Specifically, ID disruption in the presence of ACM-causing mutations causes loss of submembrane localization of protein kinase C alpha (PKCα). This in turn aberrantly activates the Hippo kinase cascade. Specifically, macrophage-stimulating protein 1/ 2 (MST1/2) phosphorylates the Large tumour suppressor kinase 1/ 2 (LATS1/2) and its scaffold protein Mps one binder 1 (MOB1). Active LATS1/2 then phosphorylates and inactivates the Yes-associated protein/ transcriptional coactivator with PDZ binding motif (YAP/TAZ) preventing it from translocating into the nucleus and binding to the transcriptional enhanced associate domain (TEAD) transcription factors (Fig. 4). Phosphorylated YAP can be driven to the IDs through binding α-catenin. However, it may also interact with the destruction complex enhancing β-catenin degradation [25].

### GSK3β Inhibitors: Successes and Failures

The importance of GSK3β in ACM pathogenesis was initially recognized through animal models. Notwithstanding the heterogeneity of studies in review, SB2 seems to show several key trends in limiting ACM-driven defects.

The first evidence stemmed from the high-throughput screening of a zebrafish model with cardiac-specific expression of *JUP*^*2157del2*^ variant, known to underlie a syndromic form of ACM in patients (Naxos disease) [3, 26]. Zebrafish ventricular CMs expressing *JUP*^*2157del2*^ showed a 70–80% reduction of the inward sodium current I_Na_ and the inward rectifying potassium current I_K1_, responsible for maintaining the resting-membrane potential. SB2 completely prevented and reversed these ionic current abnormalities [3]. These results were replicated in investigations of NRVMs transfected to express ACM-causing variants. SB2-treated ACM-NRVMs also showed restoration of localization of key proteins including plakoglobin, Cx43 and GSK3β [3, 27, 28]. Additionally, SB2 was shown to prevent and reverse redistribution of SAP97, a molecule implicated in the trafficking of Nav1.5, the potassium channel protein Kir2.1 (driving I_K1_) and plakoglobin to the membrane [3].

Later literature showed additional promising results with in vivo models. *Chelko *et al. investigated the role of GSK3β in two murine models: a *DSG2* knock-in model (*Dsg2*^*mut/mut*^) and a transgenic mutant *JUP* model (*JUP*^*2157del2*^). SB2-treated mouse strains (onset at 3 weeks for *Dsg2*^*mut/mut*^ mice and 6 months for *JUP*^*2157del2*^ mice, prior to disease manifestation) showed improved EF, reduced arrhythmic load, myocardial inflammation and fibrosis and restoration of key ID and signalling proteins (JUP, Cx43, GSK-3β, Nav1.5, SAP97) compared to vehicle-treated litter mates [4]. Of note, SB2 also significantly improved all functional parameters and reversed key protein remodelling in mice treated after disease onset [4].

Heterozygous *Dsg2*^*mut/*+^ mice do not show ACM hallmarks at rest. However, upon endurance exercise, deleterious re-distribution of JUP/Cx43 occurs in addition to arrhythmia development. SB2 administration prior to exercise prevented these defects [4]. Studies have shown efficacy of SB2 in preventing ACM-related abnormalities also in iPSC-CMs models. Specifically, iPSC-CMs derived from patients bearing *PKP2* mutations show significantly reduced I_Na_ current densities as well as subcellular redistribution of Nav1.5, restored both by SB2 and CHIR [13]. In a related study, SB2 restored Cx43 localization, electrical coupling and calcium (Ca^2+^) waveforms in mutant iPSC-CM pairs derived from ACM patients bearing *PKP2* variants [29]. This supports previous work showing that Ca^2+^ overload may contribute to the high levels of apoptosis and myocardial remodelling characterizing ACM [30].

*Hamstra *et al*.* corroborated this proposition, investigating the effects of SB2 in cytosolic Ca^2+^ handling [31]. The SERCA pump isoform, SERCA2a, maintains homeostasis by actively transporting cytosolic Ca^2+^ ions into the SR [32]. GSK3β inhibition starting at 3 weeks of age in *DSG*^*mut/mut*^ mice demonstrated an increase in SERCA2a density/activity, in contrast to vehicle-treated mice [31]. This elucidates another potential mechanism of SB2 in preventing ACM tachyarrhythmias. Likely, this may explain overall survival rates of exercising mice reported by Chelko et al.[4]. Notably, a mouse model with postnatal CM-*ANK2* deletion shows structural abnormalities reminiscent of ACM consistent with the identification of rare *ANK2* variants in ACM probands (Table 1) [33]. SB2 administration at 4 weeks of age (prior to disease manifestation) led to improved EF and reduced fibrosis in the *ANK2* mutant mice coupled with reduced levels of phosphorylated β-catenin [33]. Another mouse model expressing the *TMEM43-S358L* mutation recapitulates the human disease exhibiting CM death and severe fibrofatty replacement, preventable both by SB2 and CHIR. iPSC-CMs bearing the same mutation show marked contractile dysfunction prevented by the GSK3β inhibitor [34]. Of note, myocardial injury was independent of GSK3β pharmacological inhibitors and GSK3β levels in a mouse model of myocardial infarction highlighting differences in pathology between different heart diseases [35].

In another preclinical study, Asimaki et al. cultured buccal mucosa cells from ACM patients bearing desmosomal gene mutations. SB2 exposure of cultured cells led to restoration of Cx43/JUP signal distribution [36]. Additionally, HeLa cells expressing *JUP*^*2157del2*^ show a dramatic decrease of ID-localized Cx43 as well as marked microtubule disassembly, restored by SB2 [37].

*Giuliodori *et al. performed an in vivo cell signalling screen using pathway-specific reporter transgenes in a *DSP*-deficient zebrafish model. Three pathways (Wnt, TGF3β and Hippo/YAP-TAZ) were significantly altered, with Wnt being the most dramatically affected. Interestingly, under persistent *DSP* deficiency, the phenotype was rescuable by SB2 [38]. Furthering this work, *Celeghin *et al*.* created a *DSP* knock-out zebrafish line characterized by cardiac alterations, oedema and bradycardia at larval stages. Adult hearts showed reduced contractile structures, abnormally-shaped ventricles, myocardial layer thinning, adipocyte infiltration and disorganized desmosomes. Intensive physical training caused a global worsening of the cardiac phenotype accelerating disease progression. The mutant fish showed a dramatic decrease of Wnt signalling activation as well as Hippo/YAP-TAZ and TGFβ pathway dysregulation. SB2 administered at 1–3 days post fertilization rescued all pathway expression and cardiac abnormalities restoring the heart rhythm [39]. Although several of the studies cited above only examined the efficacy of SB2 in preventing ACM-related abnormalities, certain studies also showed that the GSK3β inhibitor can reverse disease phenotypes in varying experimental models. This is crucial given how potential clinical trials would primarily enrol already symptomatic patients with existing disease.

Caution should be exerted when evaluating trials using non-CM cell-types [36, 37]. There are also caveats when evaluating results from iPSC-CMs, as these cells demonstrate an immature phenotype. Structural variation may account for reduced ion densities, as immature CMs are not as polarized as adult variants, exhibiting different sodium channel distribution across the membrane [40]. Furthermore, results from ex vivo models may also pose differences too due to minimal inflammatory and hormonal influences compared to in vivo conditions.

Caution must also be exerted in light of a study published by *Li *et al. showing that SB2 can potentiate arrhythmic events in human cardiac slices [41]. Combined computational modelling and experimental approaches showed that the GSK3β inhibitor can decrease sodium-channel conductance and tissue conductivity underlying the observed arrhythmic phenotypes [42]. Whether or not this is due to dosing regimens, nuance at the molecular level, or modelling variance requires further discourse beyond the scope of this review. A summary of the experimental results reviewed is shown in Table 2 below.

**Table 2:** Summary of outcomes of SB2 administration in ACM preclinical models

Author and year	Model	Inhibitor	Nav1.5 dysfunction	Junctional protein distribution	Myocardial fibrosis	Other outcomes	Ref
*Asimaki *et al. (2014)	Zebrafish *JUP*^*2157del2*^	SB2	Normalized	Normalized (JUP, Cx43)	Improved	No change in GSK3β distribution	[3]
	NRVMs	SB2	Normalized	Normalized (JUP, Cx43)	N/A	I_K1_ current restored SAP97 normalized	[3]
*Hariharan *et al. (2014)	NRVMs	SB2	Unreported	Normalized (JUP, Cx43)	N/A	Normalization of shear-induced JUP remodelling	[27]
*Zhang *et al. (2014)	HeLa	SB2	Unreported	Normalized (Cx43)	N/A	Full rescue of microtubule network	[3]
*Chelko *et al. (2016)	Murine *Dsg2*^*mut/mut*^	SB2	Normalized	Normalized (JUP, Cx43)	Improved	GSK3β distribution normalized. Protective effects in exercise	[4]
	Murine *JUP*^*2157del2t*^	SB2	Normalized	Normalized (JUP, Cx43)	Improved	GSK3β distribution normalized	[4]
*Asimaki *et al. (2016)	Buccal mucosa cells	SB2	Unreported	Normalized (JUP, Cx43)	N/A	Normalization of JUP nuclear accumulation	[36]
*Giuliodori *et al. (2018)	*DSP*-deficient zebrafish	SB2	Unreported	Unreported	Improved	Restoration of Wnt/Hippo/TGFβ pathways	[37]
*Padron Barte *et al. (2019)	*TMEM*-S358L mice	SB2	Unreported	Normalized (Cx43)	Improved	GSK3β distribution/ β-catenin expression normalized	[34]
	*TMEM*-S358L mice	CHIR	Unreported	Normalized (Cx43)	Improved	GSK3β distribution/ β-catenin expression normalized	[34]
	*TMEM*-S358L iPSC-CMs	SB2	Unreported	Normalized (Cx43)	N/A	GSK3β distribution/ β-catenin expression normalized	[34]
	*TMEM*-S358L iPSC-CMs	CHIR	Unreported	Normalized (Cx43)	N/A	GSK3β distribution/ β-catenin expression normalized	[34]
*Roberts *et al. (2019)	*ANK2-KO* mice	SB2	Unreported	Unreported	Improved	Improved EF β-catenin normalized	[33]
*Khudiakov *et al. (2020)	iPSC-CMs	SB2	Normalized	Unreported	N/A	No change in GSK3β distribution	[13]
	iPSC-CMs	CHIR	Normalized	Unreported	N/A	No change in GSK3β distribution	[13]
*Hamstra *et al. (2022)	Murine *Dsg2*^*mut/mut*^	SB2	Unreported	Unreported	Unreported	Restoration of SERCA2a activity	[31]
*Kim *et al. (2023)	iPSC-CMs	SB2	Unreported	Normalized (JUP, Cx43)	N/A	Myofibrillogenesis Restoration of Ca^2+^ wavefronts	[29]
*Celeghin *et al. (2023)	*DSP*-deficient zebrafish	SB2	Normalized	Normalized	Normalized	Bradycardia rescue	[39]
*Jin *et al. (2024)	NRVMs	SB2	Normalized	Normalized (Cx43)	N/A	Restoration of conduction velocity	[28]

The table includes the experimental models used, the type of GSK3β inhibitor administered, the effect of the inhibition on the function of sodium channels, junctional protein distribution and extent of myocardial fibrosis (where relevant) as well as additional outcomes of interest

Trials utilizing CHIR in ACM models are limited [13, 34] perhaps due to its propensity to binding other kinases at high micromolar concentrations causing collateral alterations [42]. Current literature reports no use of the GSK3β inhibitors lithium and SB4 in ACM experimental models. Caution should be raised with lithium, a pervasive mood stabilizer, as it may have abnormal electrophysiologic effects by blocking sodium channels [43]. However, the GSK3β inhibitor tideglusib, has been used in phase II clinical trials for Alzheimer’s disease [44] and myotonic dystrophy [45] while the TaRGET trial, aiming to assess its efficacy in ACM patients, launched in February 2024 [5]. Of note, tideglusib is a non-ATP competitive GSK3β inhibitor. Most kinase inhibitors are designed to bind to highly homologous ATP-binding sites, which leads to promiscuity and possible off-target effects. Allosteric inhibitors, however, exhibit high specificity and selectivity minimizing potential adverse effects [46]. Consequently, the mode of action of tideglusib alone may classify it as a superior molecule of choice as a mechanistic inhibitor of ACM.

## Discussion

Aberrant activation of GSK3β was a cardinal discovery in the understanding of ACM pathogenesis. This, naturally, leads to discussions as to whether GSK3β inhibitors should be considered as a mechanism-based therapy for the disease. Although studies on ACM experimental models show encouraging data (Table 2)*,* there is skepticism over the clinical use of such inhibitors. In addition to the concerns related to all small molecules (absorption, distribution, metabolism, excretion and toxicity) [47], ACM patients would require chronic treatment and chronic inhibition of GSK3β has its own set of challenges.

Canonical Wnt signalling is a highly pertinent pathway in cancer. Wnt target genes such as c-Myc (Fig. 3), are upregulated in a number of cancers [48]. Consequently, the potential carcinogenic risks of SB2 cannot be ignored since β-catenin accumulation drives expression of such oncogenes [40]. GSK3β inhibition may also induce transcription factors coding epithelial-to-mesenchymal transition phenotypes promoting metastasis [49]. Prolonged cWnt activation can also cause cardiac hypertrophy, which may lead to pathological and prolonged hemodynamic stress [50, 51]. Data over carcinogenic or ventricular hypertrophy risk were not recorded in mice models of ACM. However, SB2 administration was over a relatively short period of time, which could have been insufficient for such phenotypes to develop [4, 13, 31, 33]. Accordingly, we identify the need for longer-term, follow-up studies of SB2 use in vivo.

It is also crucial to highlight that GSK-3β is ubiquitous in the body, and currently no in vivo study manages to restrict delivery to the heart. Methods of cardiac-specific delivery would thus be necessary to mitigate off-target effects [52]. A potential solution may lie with cardiac-targeting peptide (CTP)-expressing exosomes, which have shown successful cardiac-specific delivery in vivo [53]. However, although CTPs are non-toxic and may successfully act as a ‘vehicle’, the procedure, which involves obtaining cells from the affected individual to be cultured and genetically modified, is very expensive and time-consuming [53]. Efficacious, yet safe, CTP-driven SB2 administration is speculative. However, the TaRGET trial, which focuses on the non-ATP competitive tideglusib molecule, may well mitigate potential adverse effects [5].

A unifying weakness amongst preclinical literature is the bereft consideration regarding upstream pathways. In the trials by Celeghin et al.[39] and Giuliodori et al*.*[38]*,* confirmation of Hippo signaling deregulation was noted in mutant zebrafish, augmenting adipogenesis in ACM models. This may cause multiple pharmacologic interactions, as in the absence of Wnt-signaling, the GSK-3β destruction complex is also responsible for TAZ degradation (Fig. 4). These results urge clinicians to probe the effects of other pathways converging upstream of GSK-3β in ACM.

Lastly, the GSK3 family is comprised of two isoforms: GSK3α and GSK3β. SB2 non-specifically targets both, propelling the need for investigations over the role of GSK3α in arrhythmic development of ACM. Paradoxical interactions between the isoforms have been reported during cardiac stresses [54]. Studies in Table 2 did not differentiate this distinction.

**Fig. 5:**
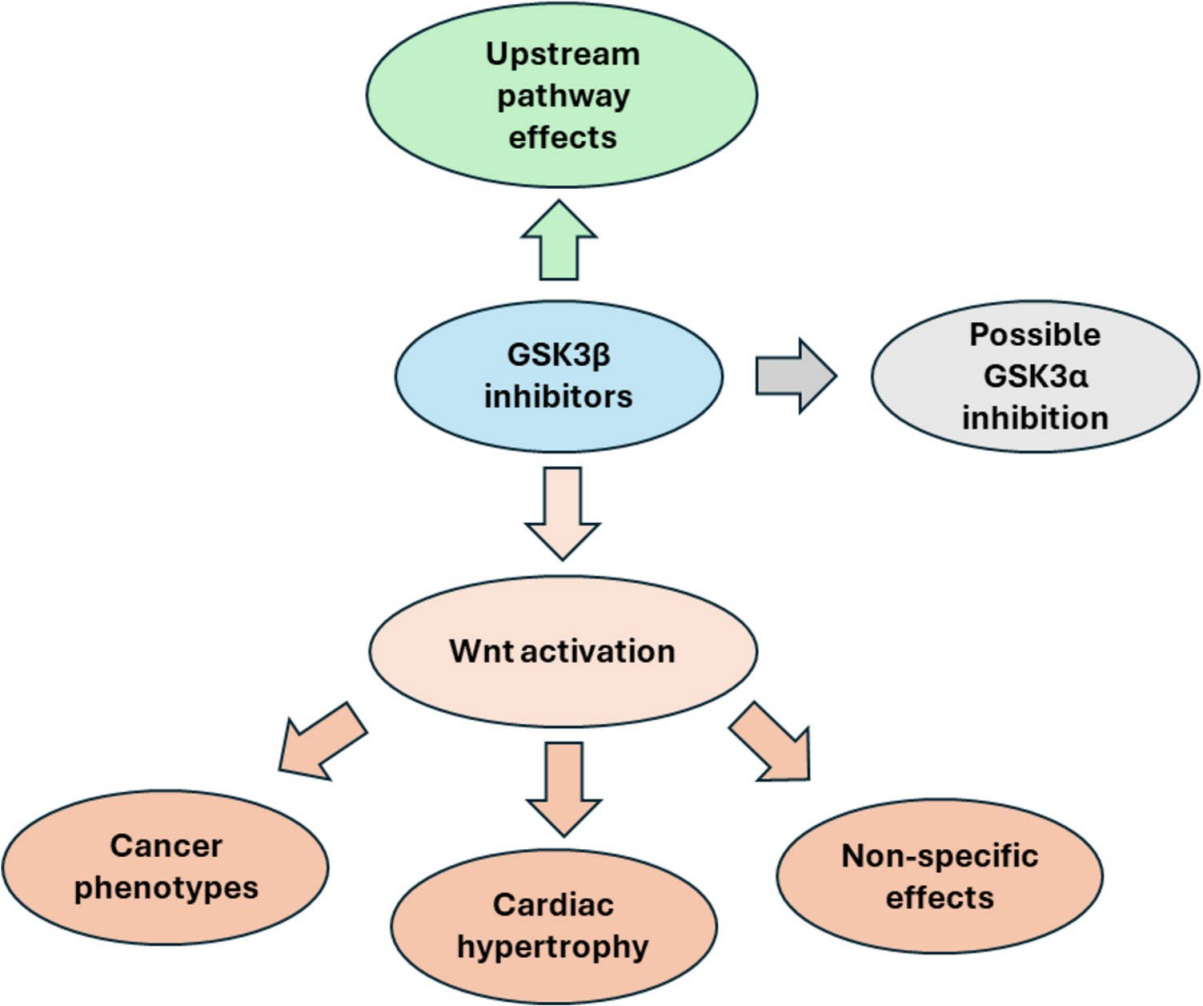
Illustration of major speculatory concerns over GSK-3β inhibitors in ACM

A summary of the concerns surrounding clinical GSK3β inhibition is presented in Fig. 5.

Wnt activation brought by GSK3β inhibition may promote cancer phenotypes, cardiac hypertrophy as well as other non-specific deleterious events. Moreover, most denoted GSK3β inhibitors may also inhibit GSK3α, adding to the list of potential off-target effects. Finally, in light of feedback regulatory mechanisms, inhibiting GSK3β may have upstream pathway effects.

Alternatively, targeting downstream effectors of GSK3β may have less adverse effects, potentially circumventing the risk of cancer. Promising studies suggest the major inflammatory nuclear factor Kappa beta (NFκB) pathway as a potential future target for ACM [55]. Multiple lines of evidence suggest that aberrant immune activation contributes to ACM phenotypes. In line with this, *DSG2*^*mut/mut*^ mice showed increased expression of inflammatory mediators normalized by the NFκB inhibitor BAY11. Additionally, NRVMs expressing *JUP*^*2157del2*^ show normalization of Cx43, JUP and GSK-3β in response to BAY11. Aberrant NFκB signaling was shown to cause contractile dysfunction and arrhythmia by mobilizing macrophages in affected heart areas, emphasizing the potential of its inhibition [56]. NFκB is directly downstream of GSK-3β, and evidence points that GSK-3β activation promotes NFκB activity [57]. However, since NFκB regulates inflammatory T-cell activation and differentiation, chronic inhibition could risk immunosuppression [57].

Notably, pharmacological GSK3β inhibition was shown to markedly improve myocardial dysfunction and prevent remodelling in a rat model of myocardial infarction through reducing NLRP3 inflammasome activation [58]. NLRP3 expression is significantly upregulated in the right ventricle of *Dsg2*^*mut/mut*^ mice. Mutant mice treated with the NLRP3-inhibitor MCC950, show normal EF and fractional shortening, develop no arrhythmias and show no cardiac fibrosis unlike vehicle-treated littermates [59]. Accordingly, this may be a mechanism through which GSK3β inhibition alleviates the myocardial structural defects characterizing ACM.

*PKP2* gene therapy has been shown to prevent and rescue ACM in mouse models bearing *PKP2* mutations [60, 61]. LEXEO Therapeutics, Rocket Pharmaceuticals and Tenaya Therapeutics have launched the first in-human studies designed to evaluate the safety and preliminary efficacy of *PKP2* administration in ACM patients bearing such mutations (https://ichgcp.net/clinical-trials-registry/NCT06109181; https://clinicaltrials.gov/study/NCT05885412; https://clinicaltrials.gov/study/NCT06228924). Unlike pharmacological inhibitors, which often require frequent administration and focus on managing symptoms and disease progression, gene therapy addresses the mutant gene and provides a long-term treatment benefit with potentially a single dose. Nevertheless, inhibitors targeting the ‘final common pathway’ of a disease, can potentially benefit all patient sub-populations regardless of the underlying genetic defect.

## Clinical Relevance

GSK3β has received much attention as a therapeutic target in ACM. While GSK3β inhibitors have shown considerable success in preclinical models, their translation clinically remains challenging. Efforts including the TaRGET trial will aim to advance this line of research. Evidence of NFκB involvement in ACM pathogenesis may facilitate the crucial identification of safer long-term treatment mechanistic targets.

## Abbreviations

ACM: Arrhythmogenic cardiomyopathy
AJ: Adherens junction
APC: Adenomatous polyposis coli
BMP: Bone morphogenic protein
BAY11: BAY117082
CCR2 +: Chemokine receptor type 2
CHIR: CHIR9902
CMs: Cardiac myocytes
Cx43: Connexin 43
cWnt: Canonical Wnt-signaling
DKK: Dickkopf
DSC2: Desmocollin-2
DSG2: Desmoglein-2
DSP: Desmoplakin
Dvl: Dishevelled
EF: Ejection fraction
Fzd: Frizzled
GJ: Gap junction
GSK3: Glycogen synthase kinase-3
IDs: Intercalated discs
ICD: Implantable cardioverter defibrillator
iPSC: Induced pluripotent stem cells
JUP: Plakoglobin
LATS1/2: Large tumour suppressor kinase 1/ 2
LEF: Lymphocyte enhancer factor
LRP5/6: Low-density receptor related protein
MOB1: Mps one binder 1
MST1/2: Macrophage-stimulating protein 1/ 2
NFκB: Nuclear factor kappa B
NRVM: Neonatal rat ventricular myocytes
PKP2: Plakophilin-2
SAP97: Synapse associated protein 97
SAV-1: Protein salvador homolog 1
SCD: Sudden cardiac death
SERCA: Sarcoplasmic reticulum Ca2 + ATPase
SB2: SB216763
SB4: SB415285
TAZ: Transcriptional coactivator with PDZ binding motif
TCF: T-cell factor
TEAD: Transcriptional enhanced associate domain
TGF-β: Transforming growth factor-beta
TMEM43: Transmembrane 43
WT: Wild type
YAP: Yes-associated protein
